# Diagnostic accuracy of ultrasonography in diagnosis of Carpal Tunnel Syndrome

**DOI:** 10.12669/pjms.40.4.8332

**Published:** 2024

**Authors:** Aamir Shaukat, Hooria Aamir, Zaheer Ahmad, Umair Ahmad

**Affiliations:** 1 Aamir Shaukat, FCPS. ***Affiliation:*** Faisalabad Medical University, Allied Hospital, Faisalabad, Pakistan; 2 Hooria Aamir, M Phil, ***Affiliation:*** Faisalabad Medical University, Allied Hospital, Faisalabad, Pakistan; 3 Zaheer Ahmad, FCPS, ***Affiliation:*** Faisalabad Medical University, Allied Hospital, Faisalabad, Pakistan; 4 Umair Ahmad, FCPS, ***Affiliation:*** Faisalabad Medical University, Allied Hospital, Faisalabad, Pakistan

**Keywords:** Carpal Tunnel Syndrome, Diagnosis, Nerve Conduction Study, Ultrasonography

## Abstract

**Objective:**

This study aimed to assess the diagnostic accuracy of Ultrasonography, considering nerve conduction study (NCS) as the gold standard diagnostic modality for carpal tunnel syndrome (CTS).

**Methods:**

A cross-sectional study was conducted at the Neurology Department of Allied Hospital, Faisalabad from August, 2020 to January, 2021. NCS and Ultrasonography of wrist were performed for diagnosis of CTS. The sensitivity, specificity, predictive value (NPV), positive predictive value (PPV), and diagnostic accuracy (DA) were calculated for the screening test (Ultrasonography of the wrist), taking NCS as the gold standard.

**Results:**

The mean age of the study population was 41.53 ± 8.80 years, with female pre-dominance (71.66%). The right hand was involved in 24(40%) patients, left hand was involved in 4(6.66%) patients, and both hands were involved in 32(53.33%) patients. Among 60 patients, ultrasonography diagnosed 56 (TP+FN) as having CTS, confirmed via nerve conduction study. Sensitivity, specificity, NPV, PPV, and DA of Ultrasonography of the wrist for CTS were 92.8%, 75%, 42.8%, 98.1%, and 91.6%, respectively.

**Conclusion:**

Based on the sensitivity and specificity, Ultrasonography may assist in diagnosing CTS.

## INTRODUCTION

The most prevalent compressive neuropathy affecting the upper limb is carpal tunnel syndrome (CTS).^1^ As the nerve travels through the carpal tunnel and under the flexor retinaculum, it becomes entrapped at the wrist. CTS refers to a collection of clinical symptoms and signs brought on by compression and a slowdown of the median nerve’s conduction velocity at the wrist.^2^ The prevalence of CTS seems to be lowest in thin and average-sized males and highest in obese women.^3^ According to estimates of the occupational prevalence of 5-15%, CTS may be more common in the workplace than in the general population.^4^ Certain occupations involving prolonged work periods, continuous exposure to vibration, and/or heat pressure are associated with a higher risk of CTS. Conditions connected to CTS include obesity, female gender, pregnancy, diabetes, rheumatoid arthritis, hypothyroidism, acromegaly, etc.^5^ A study from Saudi Arabia assessing the prevalence of CTS symptoms among female touchscreen users at Majmaah University found CTS among 34.2% touchscreen users.^6^ Further local studies from Peshawar, including butchers and dentists reported that 10.3% and 21.2% respondents reported the symptoms of CTS, respectively.^7^,^8^

In idiopathic cases, the dominant hand is more severely affected. Early diagnosis and treatment are essential since persistent compression of the median nerve can harm the nerve permanently and result in neurological impairments.^9^ The highly suggestive symptoms include pain and/or paresthesia while driving, holding phone or book, sensory disturbance in the first, second, and third digits with the splitting of the fourth digit, nocturnal awakening, hand wringing, thenar eminence weakness, and positive tinel and phalen’s signs. Patients typically seek medical attention as a result of pain and paraesthesia. Some patients express an arm-wide, diffuse, poorly localized aching.^10^ Motor fibers only become clinically engaged in more severe or advanced cases when patients report decreased dexterity due to weakness in thumb abduction and opposition.^11^

Nerve conduction studies (NCS) are fundamental components of examining CTS. They help confirm the CTS diagnosis, determine the severity, and rule out other anomalies.^12^ EMG is useful for ruling out other disorders, such as polyneuropathy, plexopathy, and radiculopathy.^13^ Although NCS is used to collect physiologic information, they have a poor sensitivity that ranges from 49% to 86% and a specificity of 95%.^14^ Imaging tests are not frequently used in the assessment for potential CTS. The median nerve’s cross-sectional area (CSA) is substantially larger in CTS patients than in controls, according to several investigations using ultrasonography.^15^

The sensitivity and specificity of this approach and the ideal CSA cutoff for the diagnosis have varied in various reports.^16^ The four studies with the highest quality in a systematic literature assessment employed diagnosis cutoffs of 8.5-10 (mm^2^).^16^ The specificities varied from 73 to 98%, and the sensitivities from 65 to 97%. Ultrasonography has numerous benefits over NCS, including accessibility, reduced cost, non-invasiveness, and quicker examination times. With nerve conduction investigations serving as the gold standard, this study aimed to evaluate the Ultrasonography diagnostic accuracy for CTS.

## METHODS

This cross-sectional study was carried out at Neurology Department, Allied Hospital, Faisalabad from August, 2020 to January, 2021. A total of (n=60) patients fulfilling the inclusion criteria were enrolled after obtaining informed consent. Patients of both genders above 18 years of age with the clinical picture of CTS (having sensory symptoms like pain paresthesia, numbness in first three and a half fingers associated with activities like writing, holding a phone, and during sleep, positive tinel or Phalen sign), were included in the study. While patients with wrist deformities and arthritis were excluded from the study.

### Ethical approval

It was obtained from the ethical review committee of Faisalabad Medical University (Ref# 1032; Dated: 24-07-2020).

NCS were performed in the neurophysiology section of the institute via Medelec Synergy T2 EMG machine by a neurologist. Ultrasonologists from Radiology Department performed ultrasonography of the wrist for CTS. Electrophysiological studies have shown that the median nerve’s motor distal latency at the abductor pollicis brevis and wrist stimulation was > 4.4 ms, the median nerve’s antidromic sensory peak latency at the second digit was > 3.5 ms, the antidromic median sensory latency and ulnar sensory latency at the fourth digit were different by more than 0.5 ms. The antidromic latency between the patients was deemed to have CTS when undergoing Ultrasonography if their increased CSA at the level of the pisiform bone was greater than 10 mm^2^.

SPSS version 22.0 was used for statistical analysis. Categorical variables were calculated using frequencies and percentages, while continuous variables (such as age) were displayed using means and standard deviations. For the screening test (Ultrasonography of the wrist), the sensitivity, specificity, NPV, PPV, and DA were computed using NCS as the gold standard.

## RESULTS

The baseline characteristics of enrolled participants is presented in Table-I. The right hand was involved in 24 (40%) patients, left hand was involved in 4 (6.66%) patients, and both hands were involved in 32(53.33%) patients.

**Table-I:** Baseline characteristics of the enrolled participants.

Variables		N=60
Age; Years		41.53±8.80
Gender	Male	17 (28.33)
	Female	43 (71.66)
Hand Involvement	Right	24(40.0)
	Left	4(6.66)
	Both	32(53.33)

Values are given as n(%) or mean±SD.

Among 60 patients, ultrasonography diagnosed 56 (TP+FN) as having CTS, confirmed via nerve conduction study. Sensitivity, specificity, NPV, PPV, and DA of Ultrasonography of the wrist for CTS were 92.8%, 75%, 42.8%, 98.1%, and 91.6%, respectively (Table-II).

**Table-II:** Diagnostic Accuracy of Ultrasonography in CTS Diagnosis.

		Gold standard (Nerve Conduction Study)		

		Positive	Negative	
Screening test (Ultrasonography of wrist)	Positive	52	1	PPV
		TP	FP	98.1%
	Negative	4	3	NPV
		FN	TN	42.8%
		Sensitivity	Specificity	DA
		92.8%	75%	91.6%

## DISCUSSION

In our study, wrist ultrasonography for the diagnosis of CTS displayed a high sensitivity, i.e., 92.8%, specificity of 75%, NPV 42.8%, PPV of 98.1%, and diagnostic accuracy of 91.6%, considering NCS as a gold standard. The CSA of the median nerve was used as a threshold for diagnosis of CTS, which is considered the most sensitive parameter for CTS on wrist ultrasonography.^17^,^18^ In one study by Azami et al., wrist ultrasonography for CTS has a sensitivity and specificity of 99.2% and 88.3%, respectively.^5^ A similar study reported that the sensitivity and specificity of wrist ultrasonography were 94.7% and 63.6%, respectively.^19^ In another study by Elnady et al., it was reported that wrist ultrasonography in CTS diagnosis using a CSA of median nerve >10 mm^2^ presented a sensitivity of 83% and specificity of 85%.^20^ In a meta-analysis by Fowler and colleagues, the composite sensitivity and specificity were 77.6% and 86.8%, respectively.^21^ Furthermore, a study comparing the effectiveness (accuracy) of Ultrasonography and NCS for diagnosing CTS confirmed equal sensitivity, specificity, and PPV, demonstrating that both diagnostic techniques successfully detected CTS. When comparing US and NCS, well-designed Standards for the Reporting of Diagnostic accuracy (STARD) studies produced similar findings.^22^,^23^

However, compared to other studies using other reference standards,^23^,^24^ neither US nor NCS could sufficiently rule out the clinical suspicion of CTS due to the study’s low negative predictive values. The variations in these findings might be due to multiple factors, including the selection criteria of patients, the gold standard for diagnosis of CTS, diagnostic methods, levels of CSA measurement, and ultrasonographic cutoff values.

Among the many favorable features of Ultrasonography compared to NCS, the first is cost-effectiveness. Ultrasonography is economical as compared to NCS and electromyography. Second, it has been claimed that it is quicker than NCS. According to Fowler et al. study of a learning curve, Ultrasonography was able to complete CTS assessments for each patient in less than 90 seconds.^25^ According to the study, reducing the number of examinations might speed up diagnosis time for each patient to 30 seconds. However, even when performed by skilled specialists, electrodiagnostic tests are expected to take about 30 minutes. Additionally, ultrasound serves as a less painful alternative, patients experience pain and discomfort during electrodiagnostic testing like electromyography. Hence the use of ultrasound as the initial diagnostic technique, particularly for patients with typical CTS has been recognized.^22^

Though Ultrasonography has its limitations compared to NCS and electromyography, CTS management is one of the major drawbacks. Unlike NCS, Ultrasonography cannot differentiate between disorders that mirror CTS. Because of this, Kwon and colleagues hypothesized that sonography could not be utilized in addition to or instead of NCS as a diagnostic tool.^26^ Because Ultrasonography is an observer-dependent examination, it has the potential to produce results and opinions that are skewed and contradictory. According to researchers, information bias may develop if the ultrasound images are assessed by several professionals using various tools.^27^ Additionally, CTS severity cannot be graded using ultrasound technology.

The practical implications of our study are significant in real world healthcare settings. The demonstrated cost effectiveness and procedural duration of Ultrasonography along with its ability to minimize patient discomfort suggest that it could play a role in influencing approaches and strategies for patient care. By recognizing these contributions, we aim to emphasize the importance of our study, in shaping the landscape of CTS diagnosis and management ultimately impacting practices and improving overall patient care quality.

### Limitations

Even though the study displayed high sensitivity and specificity of Ultrasonography for CTS diagnosis, it has some limitation. Firstly the use of Ultrasonography alone cannot be considered the standard criteria for the CTS diagnosis, specifically for advancing stages. Furthermore, the study was single-center and included only 60 individuals, which could limit how broadly the results can be applied to the Carpal Tunnel Syndrome (CTS) community.

## CONCLUSION

Ultrasonography was effective in the CTS diagnosis, showing good sensitivity and specificity for detecting patients with CTS. However, it needs to be further explored through long-term assessments. Additionally, just a few studies support the use of ultrasound alone in diagnosing CTS; nonetheless, ultrasound examination of the median nerve may be used as a screening tool in the early stages of CTS suspects. Diagnostic precision also rises with the progression of CTS’s stages and severity. Further studies are highly recommended to evaluate the outcomes of ultrasound alone and in combination with other imaging modalities for screening and diagnosing CTS.

### Authors Contribution:

**AS** and **ZA:** Have made substantial contributions to conception and design, or acquisition of data, analysis and interpretation of data.

**HA:** Participated in analysis, literature review and interpretation of data, and helped in drafting the manuscript.

**UA:** Participated in data collection, did literature search and interpretation of data.

All authors have provided final approval for publication of the manuscript and are responsible for the integrity of the study.
